# Supplementary material for: A Pilot Study on Proteomic Predictors of Mortality in Stable COPD
Source: Cells. 2024 Aug 14;13(16):1351. doi: 10.3390/cells13161351 (PMC11352814; doi:10.3390/cells13161351)
Supplement: Supplementary file 1 [file cells-13-01351-s001.zip › CELLS03127901-SM Supplementary.pdf]

SUPPLEMENTARY FIGURES

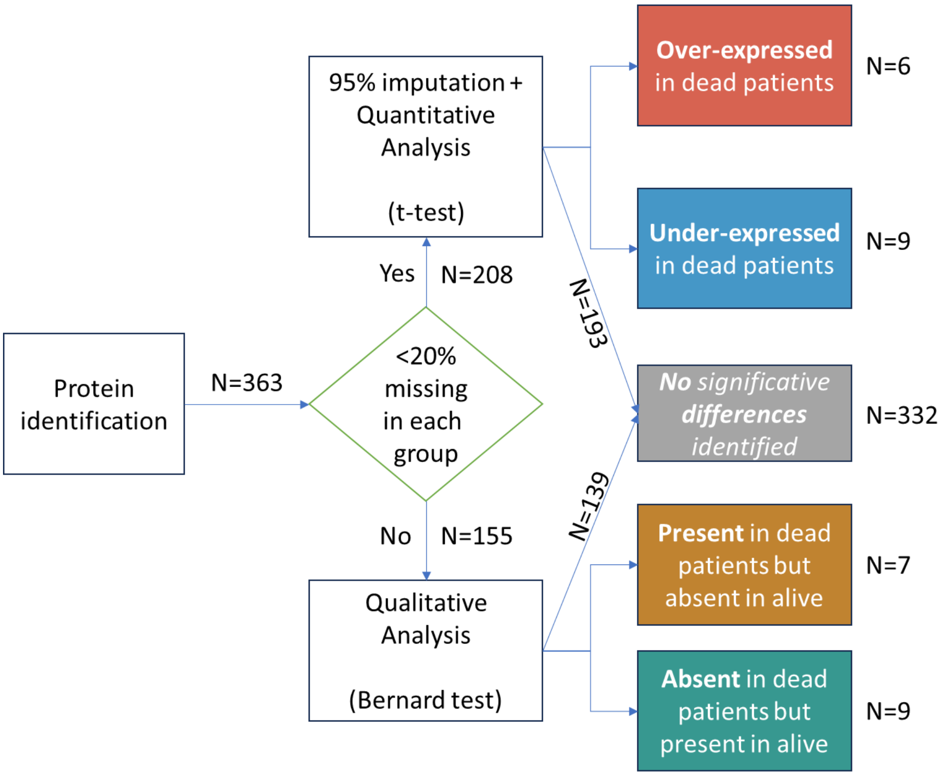

Figure S1. Flow chart with proteins, quantitative and qualitative approaches and main results. Proteins were analyzed quantitatively (over-/underrepresentation) or qualitatively (presence/ absence) using the t-test and Barnard test, respectively. Colors are equivalent to those used in the graphics of this article. Each arrow contains the number of proteins (or groups of proteins) identified

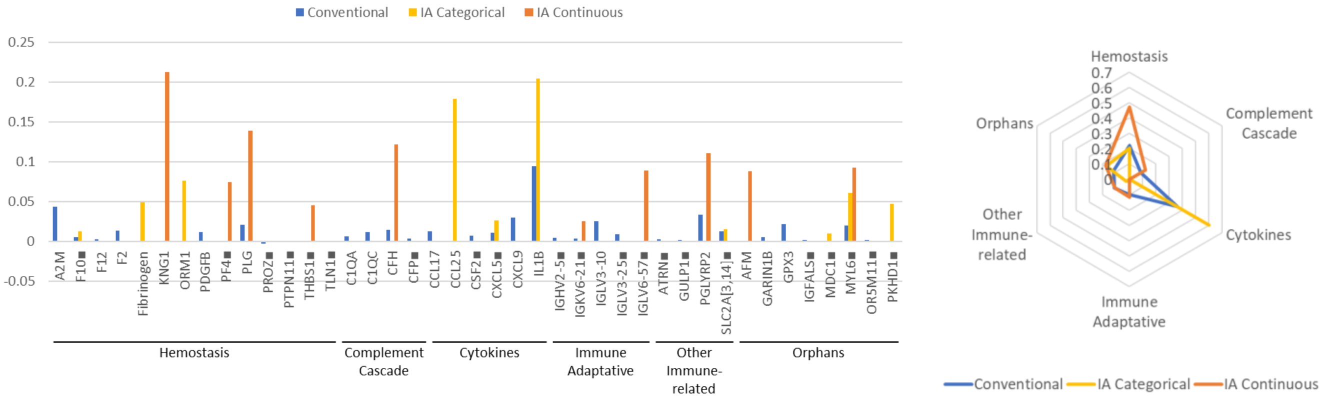

(a) (b)

Figure S2. Comparison of the importance of (A) proteins and (B) functional groups in the models. (A) Feature importances were computed using permutation with R2 scorer method for each model with Flame. Proteins are sorted by group and name. (B) To facilitate comparisons between models, a radial graph with a summation of protein importance for each functional group, normalized by the overall sum in each model, were developed. ■: qualitative variable (present/absent)

SUPPLEMENTARY DATA

Main results when patients taking antihemostatic drugs were excluded

Differentially Abundant Proteins (DAPs)

A2M, PDGFB, PLG, F10 and PTON11 remained as DAPs in the hemostasis group when patients taking antiplatelet drugs were omitted from the analysis. However, F2, F12, PROZ and TLN1 did not reach statistical significance. Similarly, if patients taking antiocoagulants become excluded, PDGFB and PLG continued in the DAP group, and pro-platelet basic protein (PPBP or CXCL7) entered as a new member. However, in this case A2M, F2, F10, F12, PROZ, PTPN11 and TLN1 did not achieve statistical significance.

Predictive Models, AI Free choice of proteins

The 10 best proteins chosen by AI for the categorical model of prediction (mortality) included in this case up to 6 proteins related to hemostasis [PLG, KNG1, coagulation factor XIII B chain (F13B), SERPINA3, and two glycoproteins (transferrin and AHSG)], with the rest belonging to either the complement cascade (CFP) or the orphan group (CD5L, a regulator of lipid synthesis, and afamin or AFM). The accuracy following validation reached 99% (coverage 88%). By contrast, the results obtained for the continuous predictive model (*days of survival*) were very poor ($Q^2 = 0.24$) with the 10 proteins chosen by the AI (CD5L, JCHAIN, SERPINA7, IL-1β, IGHV2-70D, ATRN, MYL6, MDC1, SLC2A and CCL17).

The results of the categorical prediction were also excellent if only the proteins linked to hemostasis were taken, with a validated accuracy of 94% (coverage 88%), although again the continuous prediction only obtained weak results ($Q^2 = 0.31$).

UNIPROT CODES USED FOR FUNCTIONAL CLASSIFICATION

#Id: AdaptiveImmuneSystem R-HSA-1280218

ImmuneAdaptive= ["Q13153", "Q13177", "O75914", "P16333", "Q13094", "O75791", "O43561", "P42768", "Q08881", "P19174", "P16885", "P01730", "P20036", "P01909", "P01903", "P01906", "P05538", "P04440", "P01920", "Q30154", "P79483", "P13762", "P01911", "P07766", "P04234", "P01733", "P04435", "P01850", "P01737", "P04437", "A0A0A6YYK7", "P01848", "P09693", "P20963-1", "P06239", "P43403", "Q93033", "O15117", "Q9UI08", "Q8N8S7", "P50552", "Q12913", "P08575", "P41240", "Q9Y2R2", "Q9NWQ8", "Q04759", "O95999", "O15530", "Q9BXL7", "P60484", "Q9UDY8", "Q9Y4K3", "O14920", "O15111", "Q9Y6K9", "P61088", "Q13404", "P62979", "P0CG48", "P0CG47", "P62987", "P63208", "Q13616", "Q9UKB1", "Q9Y297", "P49427", "P62837", "P51668", "P19838", "Q04206", "P25963", "Q9NYJ8", "O43318", "P27986", "O00459", "P42336", "P42338", "Q6PIZ9", "O43353", "O43242", "P60900", "Q13200", "P28066", "Q9UNM6", "P25789", "O00232", "P25788", "O00231", "P25787", "P60896", "O75832", "P25786", "Q99460", "O00487", "P62333", "P62195", "P43686", "P17980", "P35998", "P62191", "P28065", "P28062", "A5LHX3", "Q14997", "Q99436", "P28072", "Q8TAA3", "Q92530", "P61289", "P28074", "Q9UL46", "P28070", "Q06323", "O00233", "P49720", "P48556", "P49721", "P51665", "Q15008", "P40306", "Q16401", "P20618", "P55036", "O14818", "Q92835", "P17612", "P22694", "P22612", "Q13976-1", "Q684P5", "P63104", "P31946", "P47736", "Q96FS4", "P62834", "P61224", "P04049", "O95398", "Q8WZA2", "Q7LDG7", "O95267", "P20273", "P01880", "P01766", "P01768", "P01814", "P01767", "P06331", "P23083", "P01817", "P01743", "P01824", "P01763", "P01782", "P01780", "P01762", "P01825", "Q6PIL0", "P01742", "P01772", "P01764", "A2KUC3", "P01602", "P06310", "P01614", "P06315", "A0A075B6S6", "P04433", "A0A0C4DH73", "A2NXD2",

"P01601", "P01624", "A2NJV5", "P04432", "P01597", "P01619", "P01594", "P04430", "P01599", "P06312", "A0A0C4DH25", "P01593", "A0A075B6P5", "P01834", "P01615", "P01611", "P01704", "P01706", "P01709", "P01718", "P01701", "P04211", "P01705", "P01717", "P01703", "P80748", "P01714", "P01715", "P01699", "P01700", "P01721", "P0CF74", "P0DOY2", "P0DOY3", "P0CG04", "A0M8Q6", "P01871", "P11912", "P40259", "P07948", "P29350", "O00221", "Q15653", "Q04864", "P05771", "Q8IV61", "P01116-2", "P01111", "P01116-1", "P01112", "P63098", "P16298", "Q08209", "P0DP23", "Q13469", "Q12968", "O95644", "P62937", "P62942", "Q9UN19", "P15391", "P15498", "O00329", "P62993-1", "Q14643", "Q14573", "Q14571", "Q13586", "Q96SN7", "Q96D31", "P43405", "Q8WV28", "Q07889", "Q96B97", "Q06187", "P48995", "P51451", "P06241", "O43865", "Q6ZUJ8", "Q92478", "Q9NZS2", "P13612", "P26010", "Q13477-1", "Q5NV62", "Q5NV61", "Q5NV68", "Q5NV65", "Q5NV64", "Q5NV66", "Q5NV67", "Q5NV80", "Q5NV81", "Q5NV82", "Q5NV69", "Q5NV75", "Q5NV79", "Q5NV83", "Q5NV86", "Q5NV89", "Q5NV92", "Q5NV90", "Q5NV84", "Q5NV91", "Q5NV85", "Q5NV87", "P31994", "P08637", "Q9UMD9", "Q6GTX8", "O76036", "P12314", "P03452", "P04853", "P78310", "Q86YT9", "O60880", "O14796", "Q96DU3", "Q68D85", "O14931", "P06126", "P61769", "P29017", "P15813", "P29016", "Q6ISS4", "P02461", "P08123", "P02452", "Q9NQ25", "Q8IYS5", "P02458", "P35247", "Q9BZZ2", "Q96PQ1", "Q96LC7", "Q9NYZ4", "O15389", "Q96RL6", "O43699", "P20138", "Q9Y336", "Q9Y286", "Q8TD46", "P41217", "Q496F6", "Q8TDQ1", "Q08708", "Q9UGN4", "Q6UXG3", "Q6UXZ3", "A8K4G0", "Q5T2D2", "Q9NZC2", "Q9NP99", "Q6UXN2", "O95944", "Q86YW5", "Q9UHP7", "Q12918", "P25942-1", "P29965", "P05556", "P43628", "P43627", "P10321", "Q92692", "Q15762", "P01732", "P10966", "P17693", "P30511", "P13747", "P04439", "P01893", "P01889", "P40200", "P15151", "P12830", "Q96E93", "O43914", "Q14954", "P19320", "P13164", "O15181", "P60033", "P01024", "P06725", "P43631", "P43630", "Q99706", "Q13241", "P26715", "Q6UXB4", "Q9NQX5", "Q5KU26", "Q8IYJ0", "Q9UKJ1", "P43629", "O95727", "P05362", "P32942", "Q14773", "Q9UMF0", "P13598", "P05107", "P20701", "P43626", "P14151", "P28906-1", "Q8IVK1", "Q29980", "Q9BZM6", "Q29983", "Q8TD07", "Q9BZM4", "P26718", "Q9UBK5", "P14209", "Q9UKJ0", "O95971", "Q8N6C8", "O75023", "Q8N149", "A6NI73", "Q8NHJ6", "O75022", "Q6PI73", "P59901", "O75019", "Q8NHL6", "Q8N423", "P04233", "P53634", "P07858", "P43235", "P43234", "P09668", "P10619", "P14091", "P25774", "O60911", "P07339", "P07711", "Q9UBX1", "Q9UQ16", "Q05193", "P50570", "Q00610-1", "P09496-1", "O43747", "Q10567", "Q9BXS5", "Q9Y6Q5", "P61966", "P56377", "Q96PC3", "Q00610", "P09496", "O95782", "O94973", "Q96CW1", "P53680", "P63010", "Q9BUF5", "Q13885", "P68371", "Q9H4B7", "Q13509", "Q9BVA1", "Q3ZCM7", "P04350", "A6NNZ2", "Q9NY65", "Q9H853", "A6NHL2", "Q6PEY2", "P68363", "Q71U36", "Q9BQE3", "P0DPH7", "P0DPH8", "P68366", "Q13409", "O14576", "Q14204", "Q9Y6G9", "O43237", "P63167", "Q96FJ2", "Q9UJW0", "O00399", "Q9BTE1", "Q9NZ32", "P61163", "Q96KX2", "P47756", "P47755", "P52907", "Q13561", "O75935", "Q14203", "P33176", "Q12840", "Q9NSK0", "Q6P597", "Q9H0B6", "Q07866", "Q9Y496", "O15066", "O14782", "Q92845", "Q9H0H5", "O95235", "Q02241", "Q8NI77", "Q9NS87", "Q02224", "Q8N4N8", "Q99661", "O00139", "P52732", "Q9ULI4", "Q14807", "Q2VIQ3", "O95239", "Q66K46", "Q6PKD1", "O15020", "P42025", "Q96NA2", "P51149", "Q9BXW6", "P28068", "P28067", "P06340", "P13765", "P84077", "P18627", "O94979", "P55735", "Q9Y6B6", "P53992", "O94855", "O95486", "O95487", "Q15436", "P13284", "P27824", "Q99962", "Q99538", "Q13410", "P47989", "Q8WVV5", "O60437", "O00481", "P78410", "O00478", "Q6UX41", "Q6UXG8", "Q9NNX6", "Q7KYR7", "Q9UIR0", "Q92956", "Q7Z6A9", "Q06124", "Q9Y6W8-1", "O75144", "P33681", "P42081", "P10747", "P31751", "Q9Y243", "P31749", "Q92569", "Q9BPZ7", "Q6R327", "P42345", "P85299", "Q9BVC4", "Q96RU7", "Q5T1C6", "P41279", "Q99558", "P0DTC5", "P0DTC9", "P12931-1", "P07947", "P63000", "P60953", "Q9NZQ7", "Q9BQ51", "Q15116", "P16410", "P30153", "P30154", "Q15173", "Q13362", "Q15172", "Q14738", "Q16537", "P67775", "P62714", "P11021", "P30101", "P27797", "O15533", "P51572", "Q03518", "Q03519", "Q14457", "Q99570", "Q8NEB9", "Q6ZNE5", "P0DTC8", "Q6P179", "Q9NZ08", "P22897", "Q9UBG0", "Q92637", "Q9UJ71", "Q9UIQ6", "O00161", "Q99836", "P58753", "P26623", "P08571", "P16671", "O60603", "Q9Y2C9", "Q9Y6Y9", "O00206", "P02671", "P02679", "P02675", "P06702", "P05109", "P09429", "P23297", "Q15399", "P30690", "P60468", "P60059", "Q9H9S3", "P61619", "Q9BV40", "Q15836", "O75396", "Q12846", "P14598",

"Q15080", "P19878", "P13498", "P04839", "P06756", "P18084", "Q9P2P5", "Q9C037", "O15033", "Q86XT4", "Q9H0C5", "Q15034", "P62877", "Q14999", "Q92990", "Q8N3Y1", "Q9BWF2", "Q9NVR0", "Q8IYU2", "Q15345", "Q93034", "Q15369", "Q15370", "Q15751", "Q5U5Q3", "O94972", "Q8TBB1", "Q99619", "Q9BV68", "Q86XS8", "Q9BSL1", "Q5GLZ8", "Q8N7E2", "Q7Z6J8", "Q9C026", "Q96F44", "O94941", "Q9H0M0", "Q96EF6", "Q8NI29", "Q8IWV7", "Q7Z3V4", "Q9UHC7", "Q5T4S7", "Q96L50", "O15524", "O14543", "Q969Q1", "Q96A44", "Q14139", "Q05086", "Q9H4M3", "Q13049", "Q86Y13", "Q6UWE0", "Q7L5Y6", "Q13191", "Q8WVD3", "Q8N6D2", "Q96PM5", "Q15386", "Q9H2C0", "Q9ULT8", "O94822", "Q13617", "P40337", "Q9UBS8", "Q8WV44", "Q2Q1W2", "Q6Q0C0", "O94874", "Q9HCM9", "Q7Z6J0", "Q9Y576", "Q8WXK1", "Q8WXK3", "Q96DX5", "Q9H672", "Q9H765", "Q96Q27", "Q8WWX0", "A6NK59", "Q6ZVZ8", "Q9Y574", "Q96NS5", "Q8WXI3", "Q8WXK4", "Q8WXH4", "Q9NWX5", "Q8WXJ9", "Q9Y575", "Q9UBF6", "Q6ZNA4", "Q5VTB9", "Q96BH1", "Q9H4P4", "O60260", "P78317", "O95714", "Q9HCE7", "Q05516", "Q9P2K6", "C9JR72", "Q9P2N7", "Q14145", "Q53GT1", "Q9Y2M5", "Q96KE9", "Q96PQ7", "O60662", "Q9H0H3", "Q86V97", "Q9UJP4", "Q9P2J3", "O95198", "Q96MP8", "Q8WVZ9", "Q9UH77", "Q8NC69", "Q8NFY9", "Q13618", "Q9UNE7", "O60291", "Q969K3", "Q5XPI4", "Q9Y252", "Q96J02", "Q9UJX3", "Q9BS18", "Q9UJX4", "Q8NHZ8", "Q12834", "Q9UM11", "Q9NYG5", "Q13042", "Q9H1A4", "Q9UJX6", "Q9UJX5", "P30260", "Q9UJX2", "Q9UM13", "P46934", "O43164", "Q5T447", "Q8NG27", "O95376", "Q9Y4B6", "Q86WT6", "Q7Z6E9", "Q9Y508", "Q96PU5", "Q63HN8", "Q8ND25", "Q8NHG8", "Q9NQ86", "Q9H9P5", "Q9UKA2", "Q9NVW2", "Q96BD6", "Q8TC41", "Q9NV58", "Q8WY64", "Q9BYM8", "Q5U5R9", "Q8IVU3", "Q9Y6I7", "Q17RB8", "Q9Y4L5", "Q14669", "Q5XUX1", "Q9UKT7", "Q8WWI1", "Q9UKA1", "Q96IG2", "Q969U6", "Q96ME1", "Q96CD0", "Q9H469", "Q9Y3I1", "Q9UK22", "Q8NEE6", "Q9UK96", "Q8TB52", "Q86XK2", "Q8NCQ5", "Q8TF61", "Q5XUX0", "Q9UK97", "Q9UJT9", "Q8N461", "Q9NXK8", "Q9UH90", "Q6X9E4", "Q8NEZ5", "Q5XX13", "Q969H0", "P41002", "P57775", "Q8N1E6", "Q9UKT6", "Q13309", "Q6PCT2", "O94952", "Q9UKT5", "Q9NRD1", "Q6P050", "Q9UKT8", "Q969P5", "Q7Z6Z7", "O43255", "Q9HAU4", "Q8IUQ4", "Q9UII4", "Q6ZMZ0", "Q8TDB6", "Q96AX9", "Q8IWV8", "Q7Z419", "P19474", "P29144", "Q13867", "P55786", "P52888", "Q969T4", "Q969M7", "Q5VVX9", "Q8N2K1", "P63146", "Q96B02", "P62256", "Q9C0C9", "P60604", "Q9H832", "P61081", "Q8WVN8", "P61086", "Q9Y2X8", "Q16763", "P41226", "P61077", "Q9Y385", "Q15819", "O00762", "P62253", "Q96LR5", "Q7Z7E8", "P68036", "Q712K3", "P51965", "O14933", "P49459", "O95352", "Q9GZZ9", "A0AVT1", "P22314", "Q8TBC4"]

#Id: ComplementCascade R-HSA-166658.6

ComplementCascade = ["P0C0L4", "P0C0L5", "P06681", "P01024", "P00751", "P27918", "P01031", "O00187-1", "P48740", "P11226", "Q15485", "O00602", "O75636", "Q9BWP8", "Q9Y6Z7", "Q6PIL0", "P01742", "P01814", "P01766", "P01772", "P01782", "P01817", "P01767", "P01825", "P01824", "P01780", "P01762", "P01764", "P23083", "P01763", "P01743", "P06331", "A2KUC3", "P01768", "P01861", "P01857", "P01860", "P01859", "Q5NV62", "Q5NV61", "Q5NV68", "Q5NV65", "Q5NV64", "Q5NV66", "P01701", "Q5NV67", "Q5NV80", "P01709", "P01715", "Q5NV81", "P01717", "Q5NV82", "Q5NV69", "P01700", "Q5NV75", "P01721", "P01714", "Q5NV79", "Q5NV83", "Q5NV86", "P80748", "Q5NV89", "P01703", "Q5NV92", "Q5NV90", "Q5NV84", "P04211", "Q5NV91", "Q5NV85", "Q5NV87", "P01699", "P01705", "P01718", "P01706", "P01704", "P0CF74", "P0CG04", "P0DOY2", "P0DOY3", "A0M8Q6", "P01834", "P06312", "P01619", "P01615", "P01601", "P01614", "A0A0C4DH25", "A2NXD2", "P01611", "P01593", "P06310", "P01624", "P04432", "A0A0C4DH73", "P04433", "A0A075B6S6", "P01597", "P01599", "A0A075B6P5", "P04430", "P01602", "P06315", "P01594", "A2NJV5", "P00736", "P09871", "P02745", "P02747", "P02746", "P02741", "P51124", "P00746", "P20851", "P04003", "P15529", "P05155", "P04004", "P13671", "P10643", "P07225", "P05156", "Q02985", "P08603", "P08246", "P00734", "P02748", "P07358", "P07360", "P07357", "P13987", "Q03591", "Q9BXR6", "Q92496", "P36980", "P21730", "P10909", "Q16581", "P08174", "P17927", "Q96IY4", "P22792", "P15169", "P20023", "Q9P296", "P60033", "P15391"]

#Id: Hemostasis R-HSA-109582.5

Hemostasis = ["Q16621", "Q9ULX9", "O15525", "O60675", "P05015", "P01562", "P01568", "P05014", "P01567", "P32881", "P01566", "P01569", "P01563", "P01570", "P01571", "P05013", "P01574", "P10914", "P14316", "O15315", "O43502", "O14492", "Q9UQQ2", "Q9NRF2", "O60674", "Q9UKL0", "Q9P0W2", "Q92769", "Q13547", "Q96BD5", "O60341", "Q9H1K0", "Q9NZN4", "Q9NZN3", "Q9H4M9", "Q9UIJ7", "P10242", "P04637", "Q96ST3", "Q8TDN4", "P30291", "P24941", "P47756", "P47755", "P52907", "P60709", "P45973", "P68431", "Q71DI3", "P84243", "Q8IWA4", "O95140", "Q5VZK9", "P20339", "Q9NRW7", "Q8IX07", "Q8WW38", "P23769", "Q92908", "Q9BWX5", "P15976", "P23771", "P43694", "Q9BW19", "Q9H0H5", "O95235", "Q02241", "Q9HAQ2", "Q9Y496", "O15066", "O14782", "Q92845", "B7ZC32", "O60333", "O75037", "Q96Q89", "Q6ZMV9", "O43896", "Q86VH2", "Q96L93", "Q12756", "Q2TAC6", "Q9NQT8", "Q7Z4S6", "Q86Y91", "Q2KJY2", "Q96FN5", "Q96AC6", "Q9UIL4", "P33176", "Q12840", "Q9NSK0", "Q6P597", "Q9H0B6", "Q07866", "Q8NI77", "Q9NS87", "Q02224", "Q8N4N8", "Q99661", "O00139", "P52732", "Q9ULI4", "Q14807", "Q2VIQ3", "O95239", "Q66K46", "Q6PKD1", "Q9BUF5", "Q13885", "P68371", "Q9H4B7", "Q13509", "Q9BVA1", "Q3ZCM7", "P04350", "A6NNZ2", "Q9NY65", "Q9H853", "A6NHL2", "Q6PEY2", "P68363", "Q71U36", "Q9BQE3", "P0DPH7", "P0DPH8", "P68366", "Q13572", "Q8TDZ2", "P69892", "P02042", "P02100", "P68871", "P69891", "P13861", "P31323", "P10644", "P31321", "P17612", "P22694", "P22612", "O43572", "Q92667", "Q00535", "P00519", "Q9BTV7", "P60953", "P63000", "Q9H7D0", "Q8IZD9", "Q96N67", "Q92608", "Q96BY6", "Q8NF50", "Q8N1I0", "Q96HP0", "Q9BZ29", "Q14185", "Q5JSL3", "Q15652", "P04049", "P63104", "P21333", "P04275", "P08123", "P02452", "P14770", "P07359", "P13224", "P40197", "P12931-1", "P27986", "P21731", "P62879", "Q9HAV0", "O14775", "P16520", "P62873", "Q9UK08", "O14610", "P63211", "O60262", "Q9P2W3", "P63215", "P50151", "P61952", "P50150", "P63218", "Q9UBI6", "P59768", "Q14344", "P21731-3", "P21731-2", "Q13685", "P29992", "P30679", "P50148", "O95837", "P47900", "Q16539", "P47712", "Q9H244", "P08754", "P63096", "P04899", "A8MTJ3", "P62937", "Q01518", "P23528", "P07737", "O15439", "Q06481", "Q96C24", "Q6YHK3", "P05106", "P08514", "P16284", "O60245", "P16109", "P21926", "O75167", "Q9UHQ9", "P16671", "Q96JJ7", "Q6ZUX7", "Q5SQ64", "Q9BYX7", "Q8WZ42", "P18206", "P55145", "P08758", "P0DP23", "Q15833", "P00441", "O75083", "Q5JVS0", "O94919", "P37802", "Q9ULD4", "Q9Y490", "P08567", "P08962", "P13473", "O00194", "P02787", "P02647", "Q8WXD2", "Q7L3B6", "P02749", "Q13103", "P49908", "P29622", "Q06033", "P35625", "Q08380", "Q92520", "Q99969", "Q16610", "Q14624", "P56202", "P05452", "Q9NUQ9", "Q8NBF2", "P01133", "P05155", "P02776", "P01042", "P05019", "P01344", "P05121", "P00747", "P04196", "P09486", "O43707", "P12814", "P35609", "P00746", "P02775", "Q13201", "P01137", "P61812", "P10600", "O00292", "P02768", "P07225", "P14210", "P12259", "P08697", "P02751", "P62328", "P00488", "P01009", "P05067", "P01023", "P02679", "P02671", "P02675", "P01033", "P10124", "P04075", "P10909", "Q9Y6I9", "P07996", "P00451", "Q6UXV4", "Q14393", "O14498", "Q86UX7", "P01011", "P02763", "P02765", "P04085-1", "P01127", "Q9NTK5", "P19652", "Q8NBM8", "Q8NBX0", "P15692", "P49767", "O43915", "P49765", "Q9UNF1", "Q9UEU0", "Q9BX10", "Q9NXH8", "P04217", "O00391", "P07602", "Q9BWS9", "O43852", "P11021", "P05129", "P17252", "P05771", "O00186", "Q12846", "Q96RI0", "O00254", "Q03113", "P25116", "P32121", "P27361", "P28482", "P00734", "P49407", "P40238", "P40225", "P18825", "P18089", "P08913", "Q7Z5R6", "P62834", "P61224", "P12931", "Q05397", "P41240", "Q7LDG7", "O95267", "P62993-1", "Q07889", "O95398", "Q8WZA2", "P18031", "O15530", "P31749", "P43405", "P29353", "P56945", "P46108", "P06241", "P30273", "Q9HCN6", "P07948", "P16885", "Q86YL7", "Q9P126", "P52735", "P15498", "Q9UKW4", "Q13094", "P61586", "P84095", "Q05513", "P06239", "O43561", "P29350", "Q06124", "O95866", "P15153", "P42338", "Q92569", "O00459", "Q8WYR1", "Q5UE93", "P48736", "P42336", "P62745", "P24723", "Q05655", "Q04759", "Q02156", "Q9Y4D2", "Q8NCG7", "Q9BV23", "Q8N2K0", "Q99685", "Q16760", "Q5KSL6", "O75912", "P49619", "P52824", "Q86XP1", "Q13574", "P52429", "Q9Y6T7", "P23743", "Q14643", "Q14573", "Q14571", "Q13507", "Q9HCX4", "Q9Y210", "P00750", "Q03405", "P00749", "P07093", "P50452", "P35237", "P05120", "P07355", "P60903", "Q76LX8", "P17301", "P05556", "O75578", "P56199", "Q99487", "P56373", "Q99571", "Q99572", "Q9UBL9", "P51575", "O15547", "Q93086", "P43119", "Q5JWF2", "P63092", "P09769", "Q14114", "P04114",

"P30153", "P30154", "Q15173", "Q13362", "Q15172", "Q14738", "Q16537", "P67775", "P62714", "P23634", "Q01814", "Q16720", "P20020", "O14983", "Q93084", "P16615", "P32418", "P57103", "Q9UPR5", "P30626", "Q96SN7", "Q96D31", "Q13586", "P29474", "P35228", "P29475", "O75343", "Q02153", "P33402", "Q02108", "Q13976-1", "Q13237", "Q86W47", "Q9NPA1", "Q9Y691", "Q16558", "Q12791", "Q9Y6F6", "P54750", "Q01064", "O00408", "Q9HCR9", "Q9Y233", "O76083", "O76074", "P01591", "Q6PIL0", "P01742", "P01814", "P01766", "P01772", "P01782", "P01817", "P01767", "P01825", "P01824", "P01780", "P01762", "P01764", "P23083", "P01763", "P01743", "P06331", "A2KUC3", "P01768", "P01877", "P01876", "Q5NV62", "Q5NV61", "Q5NV68", "Q5NV65", "Q5NV64", "Q5NV66", "P01701", "Q5NV67", "Q5NV80", "P01709", "P01715", "Q5NV81", "P01717", "Q5NV82", "Q5NV69", "P01700", "Q5NV75", "P01721", "P01714", "Q5NV79", "Q5NV83", "Q5NV86", "P80748", "Q5NV89", "P01703", "Q5NV92", "Q5NV90", "Q5NV84", "P04211", "Q5NV91", "Q5NV85", "Q5NV87", "P01699", "P01705", "P01718", "P01706", "P01704", "P0CF74", "P0CG04", "P0DOY2", "P0DOY3", "A0M8Q6", "P01834", "P06312", "P01619", "P01615", "P01601", "P01614", "A0A0C4DH25", "A2NXD2", "P01611", "P01593", "P06310", "P01624", "P04432", "A0A0C4DH73", "P04433", "A0A075B6S6", "P01597", "P01599", "A0A075B6P5", "P04430", "P01602", "P06315", "P01594", "A2NJV5", "Q8WWV6", "P78380", "Q04881", "Q04883", "Q04875", "Q04880", "P09888", "Q04884", "Q04885", "Q05033", "Q05034", "P11296", "Q04879", "Q04877", "P11297", "Q04874", "Q04876", "Q04878", "Q04882", "P06731", "P13688", "P40199", "P40198", "P78310", "Q86YT9", "P04233", "P14174", "Q15389", "Q02763", "P01116-2", "P01111", "P01116-1", "P01112", "Q14451", "Q14449", "O60496", "Q9Y264", "O15123", "P07204", "P04070", "Q9UNN8", "P10720", "P16422", "P13612", "P07947", "Q92835", "P06756", "P19174", "P23229", "P26006", "P35613", "P16150", "Q03135", "Q13356", "P54709", "P14415", "P05026", "P20916", "P32004", "P03956", "P53985", "O15427", "O95907", "P08195", "Q9NS82", "P82251", "Q9UM01", "Q9UHI5", "Q01650", "Q92536", "Q9UPY5", "Q9BX67", "Q14242", "P16581", "Q96AP7", "P14151", "Q92896", "P16070", "Q9Y624", "P11215", "P05107", "P57087", "Q9BZW8", "P09326", "P08648", "P31997", "Q8TCZ2", "P14209", "Q9U4X0", "Q76NM5", "P19214", "P04921", "P02724", "P06028", "P20701", "Q9UIB8", "P06729", "P19256", "Q9NP99", "Q12866", "P20702", "Q8N6Q3", "Q9P1W8", "P78324", "Q08722", "O75056", "P18827", "P34741", "P31431", "P35052", "Q00889", "Q15238", "Q9UQ74", "Q00887", "Q00888", "P11465", "Q13046", "Q16557", "Q9UQ72", "P11464", "O14763", "O00220", "Q9UBN6", "Q9UKI3", "P12018", "P15814", "P01871", "P41732", "Q9NRD5", "P13726", "P08709", "P00742", "P00740", "P10646", "P03951", "P00748", "P03952", "P05546", "P01008", "P05154", "Q07021", "P42785", "P05160", "P24158"]

Id: ImmuneInnate R-HSA-168249.10

ImmuneInnate = ["Q9NR97", "Q9NYK1", "Q9NR96", "O15455", "Q9H1C4", "P43235", "P07858", "P25774", "P07711", "Q99538", "P14625", "Q9BT09", "O60911", "Q9Y4K3", "P51617", "Q8N2H9", "Q96FA3", "Q9HAT8", "P61088", "Q13404", "P62979", "P0CG48", "P0CG47", "P62987", "Q9Y6K9", "O15111", "O14920", "Q9NWZ3", "Q9BXR5", "P04949", "O60602", "Q99836", "O43187", "Q00653", "P19838", "Q04206", "P05067", "P80511", "P04271", "P09429", "P0DJI8", "Q15109", "Q70UQ0", "Q9UMW8", "Q15048", "Q86WI3", "P54578", "Q12933", "O75113", "Q14790", "P04637", "Q86UT6", "O43318", "Q15750", "Q96QP1", "Q96CG3", "Q9NYJ8", "Q8N5C8", "P25963", "Q15653", "Q9HC29", "Q9Y239", "O43353", "P0DTC9", "Q9NYR9", "Q9NYS0", "O14733", "P45985", "P45984", "P53779", "P45983", "Q16644", "P49137", "Q16539", "Q15759", "P46734", "P52564", "Q14738", "P62714", "P67775", "P30153", "P30154", "P28482", "Q13164", "P27361", "O75582", "P19419", "Q15349", "Q15418", "P51812", "Q06413", "Q02078", "Q13115", "P51452", "Q16828", "Q16829", "Q8IV63", "P16220", "P18846", "P05412", "P01100", "P15336", "P41279", "Q8NFZ5", "Q02750", "P63208", "Q13616", "Q9UKB1", "Q9Y297", "Q13233", "Q9BQ95", "Q6SZW1", "P08571", "O00206", "Q9Y6Y9", "Q86XR7", "Q8IUC6", "Q13546", "Q13158", "Q9Y572", "Q13114", "Q92844", "Q14164", "Q9UHD2", "Q14653", "Q92985", "Q06124", "P61077", "P62837", "P51668", "Q13490", "Q13489", "P18428", "P11215", "P05107", "P58753", "P26623", "P16671", "O60603", "Q9Y2C9", "P02671", "P02679", "P02675", "P06702", "P05109", "P23297", "Q15399", "P30690", "Q06187", "Q6IA17", "O15524", "Q9Y616", "O95711", "Q99467", "Q8IWL2", "Q8IWL1", "P35247", "P16885", "Q05193", "P50570", "Q9UQ16", "P29074", "P17213", "Q99570",

"Q8NEB9", "Q9H1K0", "Q15075", "P04114", "P57764", "O60443", "P0C0L4", "P0C0L5", "P06681", "P01024", "P00751", "P27918", "P01031", "O00187", "P48740", "P11226", "Q15485", "O00602", "O75636", "Q9BWP8", "Q9Y6Z7", "Q6PIL0", "P01742", "P01814", "P01766", "P01772", "P01782", "P01817", "P01767", "P01825", "P01824", "P01780", "P01762", "P01764", "P23083", "P01763", "P01743", "P06331", "A2KUC3", "P01768", "P01861", "P01857", "P01860", "P01859", "Q5NV62", "Q5NV61", "Q5NV68", "Q5NV65", "Q5NV64", "Q5NV66", "P01701", "Q5NV67", "Q5NV80", "P01709", "P01715", "Q5NV81", "P01717", "Q5NV82", "Q5NV69", "P01700", "Q5NV75", "P01721", "P01714", "Q5NV79", "Q5NV83", "Q5NV86", "P80748", "Q5NV89", "P01703", "Q5NV92", "Q5NV90", "Q5NV84", "P04211", "Q5NV91", "Q5NV85", "Q5NV87", "P01699", "P01705", "P01718", "P01706", "P01704", "P0CF74", "P0CG04", "P0DOY2", "P0DOY3", "A0M8Q6", "P01834", "P06312", "P01619", "P01615", "P01601", "P01614", "A0A0C4DH25", "A2NXD2", "P01611", "P01593", "P06310", "P01624", "P04432", "A0A0C4DH73", "P04433", "A0A075B6S6", "P01597", "P01599", "A0A075B6P5", "P04430", "P01602", "P06315", "P01594", "A2NJV5", "P00736", "P09871", "P02745", "P02747", "P02746", "P02741", "P51124", "P00746", "P20851", "P04003", "P15529", "P05155", "P04004", "P13671", "P10643", "P07225", "P05156", "Q02985", "P08603", "P08246", "P00734", "P02748", "P07358", "P07360", "P07357", "P13987", "Q03591", "Q9BXR6", "Q92496", "P36980", "P21730", "P10909", "Q16581", "P08174", "P17927", "Q96IY4", "P22792", "P15169", "P20023", "Q9P296", "P60033", "P15391", "Q9BY15", "O75165", "P16070", "P16284", "Q13576", "Q01469", "P25024", "Q5T9A4", "P21462", "P63000", "P25025", "Q9NP90", "Q9Y336", "O60610", "Q9Y6G9", "P10321", "O75022", "O43914", "O00241", "P46940", "P55160", "Q9NP72", "P15291", "P11717", "Q7L591", "Q86UQ4", "P11169", "P51575", "Q8WTT0", "P17931", "P43353", "P61018", "Q969Q5", "Q7Z7N9", "Q9HBH0", "Q8N138", "P35237", "P61586", "P20336", "P51149", "P20340", "Q5JV73", "P48960", "P84095", "P61020", "P12318", "Q8NHG7", "P61026", "Q96D96", "Q86WV6", "Q13636", "P14151", "P01889", "P25105", "P08575", "P48595", "O75015", "Q93050", "P19256", "O15389", "P08473", "Q9NPY3", "P20702", "P15144", "P53618", "O94973", "P13688", "Q68CP4", "Q9Y2Q0", "Q13393", "P98196", "Q10588", "O00264", "Q9NY25", "Q86Y34", "Q5QGZ9", "Q08722", "Q8WXI8", "Q8N6Q3", "Q9NV96", "Q969X1", "P00749", "Q9BV40", "Q8N6C8", "Q8NG11", "Q9H3Z4", "Q9Y5K8", "P04839", "P27105", "Q12913", "P22732", "Q5T4S7", "P13498", "P31997", "P78325", "O75907", "O14975", "O14672", "Q07065", "Q7Z6P3", "P62834", "O15120", "P24071", "Q9NPA2", "O15126", "Q03405", "P20138", "Q96AX2", "P61225", "P25090", "O94886", "Q7Z403", "Q8N697", "Q9Y2Q5", "Q9UHA4", "P20701", "P40198", "P19397", "B6A8C7", "Q9BXS5", "P30926", "Q6ZQN7", "P20333", "Q14165", "O94804", "P06756", "Q6IAA8", "O00161", "P30519", "Q13303", "Q9NQS5", "P60880", "P23467", "Q4KMQ2", "Q8IX19", "Q9NX76", "Q8NHP6", "Q8IWA5", "Q8TF62", "O94759", "P78380", "Q6GTX8", "O60784", "Q96HJ5", "O15121", "Q8NET5", "O95415", "P42785", "O15260", "P49768", "P27449", "Q9P0L0", "P11279", "P01833", "Q99829", "O75131", "Q9Y2G3", "P48060", "P08962", "P39656", "Q96BM9", "P13473", "Q8NF37", "O43759", "P10620", "O95721", "Q92542", "Q9H0U3", "P51148", "O95497", "P01893", "O95298", "P61224", "P07602", "P10253", "O95716", "P15309", "Q12912", "P34810", "P40199", "Q10589", "O00462", "P14174", "P18206", "Q14314", "P68402", "Q9Y315", "O75874", "P09211", "Q14974", "P08238", "P25787", "Q13162", "P11142", "O60234", "P16278", "P14780", "P00491", "P63172", "P15586", "Q96G03", "P0DMV9", "P08236", "P68104", "O00571", "P02794", "P22392", "Q14764", "P04040", "Q99436", "P04075", "P36871", "Q9P0J7", "P52790", "P68871", "P17066", "P13639", "Q12905", "P18669", "P98066", "P06737", "P06744", "Q92882", "Q9UJU6", "Q8WVQ1", "P09960", "P0DMV8", "P35998", "O00487", "P35573", "Q13442", "O00232", "P01009", "P17980", "P04264", "P55072", "P61160", "Q16769", "P37108", "O00231", "Q13200", "P16035", "P07900", "Q7Z6Z7", "P62699", "P46976", "P06396", "Q15008", "P09972", "P32320", "P41218", "P51665", "P20839", "Q9UNM6", "O43242", "P01034", "Q9P000", "P04080", "P62937", "P14618", "Q14019", "Q9H0B8", "P15848", "Q86VP6", "P13716", "Q9UNP9", "Q13510", "P50990", "Q9UBI1", "P02750", "Q14004", "P42025", "Q9UBR2", "P13798", "Q8TD55", "O15511", "P07339", "Q01432", "P04217", "Q9BU76", "Q9NZ32", "P53396", "Q9Y376", "P17858", "P14923", "P07384", "P12268", "P13727", "P28066", "P09668", "O95352", "P20618", "Q9NZT1", "P67870", "P09917", "O14727", "P12956", "Q9UBW5", "P15289", "Q13217", "P10153", "O75629", "P05089", "P02792", "P07355", "P20160", "Q01518", "P08311", "Q96CS3",

"P01011", "P19652", "Q9NZK5", "P42685", "P59665", "Q9H0E2", "P28799", "P53990", "Q86YZ3", "P34059", "Q9NZF1", "P78371", "Q92619", "Q9HD89", "P07478", "Q7Z4R8", "Q8N1F8", "Q70J99", "Q9Y2J8", "O00754", "O00584", "P00387", "Q9H410", "P68371", "Q05655", "Q99536", "P24158", "P50395", "P29508", "Q5JS37", "Q9UQ80", "Q6XQN6", "P31151", "P12724", "Q92820", "P04066", "Q9BTY2", "Q14204", "P20933", "Q9UHL4", "Q9ULZ3", "P28676", "Q9Y5Z4", "P11216", "P61916", "P06280", "P17900", "Q8NBS9", "P02766", "P05164", "Q9H7Z7", "Q9Y5R8", "O00560", "P07437", "Q99460", "P07686", "Q9BRF8", "P10619", "P53634", "P61626", "P30041", "P03973", "P09341", "Q9Y251", "P02788", "O75594", "Q96NW7", "P61769", "Q8N2G8", "Q99519", "P26022", "P00738", "Q13231", "Q99439", "Q13813", "P22894", "O15078", "Q7L576", "P41439", "P12838", "Q9BS26", "Q9BSW2", "P49184", "Q9Y2Y8", "Q92608", "P09110", "P51159", "P19801", "Q8IYS5", "P80188", "P20061", "O00391", "Q8IUR7", "Q9NQR4", "P36222", "Q6UX06", "P54108", "P02763", "P49913", "O00764", "P29350", "P02765", "P11678", "P25815", "P30740", "P31949", "Q13464", "P09769", "Q9BRR9", "P07741", "P13010", "P01111", "P30273", "O43451", "Q08ET2", "Q9UGN4", "P78324", "P49279", "P63167", "O95210", "Q8N423", "Q9Y3L5", "O75787", "P61106", "Q9H1C7", "Q6ZNJ1", "O94856", "Q96P63", "Q9Y6X5", "Q13488", "P15924", "Q13835", "Q7Z3J2", "Q08554", "Q02413", "Q8IV04", "Q92932", "P02775", "Q5D862", "P35030", "Q9H8H3", "Q7Z5G4", "P29466", "P52616", "P06179", "P41785", "Q9NPP4", "Q99572", "Q96RD7", "Q96P20", "P09616", "Q9Y2Z0", "P10599", "Q9H3M7", "P09601", "O43586", "O15553", "Q9C000", "P10415", "Q07817", "O14862", "P21580", "Q96J02", "Q9NQC7", "P53778", "O15264", "Q13685", "Q9H257", "P42575", "P55211", "P49662", "O95786", "Q8IUD6", "Q14258", "Q9C037", "Q9BYX4", "Q7Z434", "P05015", "P01562", "P01568", "P05014", "P01567", "P32881", "P01566", "P01569", "P01563", "P01570", "P01571", "P05013", "P01574", "Q09472", "Q92793", "Q9BRV8", "Q92851", "O94826", "P0DTD2", "P0DTD1", "P0DOE9", "P0DTC5", "Q13526", "P05161", "Q15366", "O14933", "P61086", "Q96EQ8", "Q96G74", "Q9H1Y0", "O94817", "Q9NWF9", "Q86VP1", "P41226", "Q9UII4", "Q96C10", "Q3LXA3", "P22079", "Q8N8Y2", "P61421", "Q9Y487", "Q9HBG4", "Q99437", "O15342", "Q8NHE4", "Q16864", "Q9UI12", "Q8NEY4", "P21283", "P36543", "Q96A05", "Q96LB4", "O95670", "O75348", "P15313", "P21281", "P38606", "P15153", "P14598", "Q15080", "P19878", "P29474", "P35228", "P29475", "Q5EBL8", "Q04656", "O00244", "P22749", "P41797", "P46587", "P15516", "P15515", "Q6UW15", "Q06141", "P14555", "O95925", "P04279", "Q8TDE3", "Q93091", "Q9H1E1", "Q96LB9", "Q96LB8", "Q969E1", "P59827", "Q8NFQ5", "Q8N4F0", "Q8TDL5", "Q9NP55", "Q96DR5", "P81605", "Q86SG5", "P10645", "Q96PD5", "Q8WWA0", "P19957", "P59666", "Q01523", "P01730", "Q01524", "P04578", "P52961", "Q30KP8", "Q9H1M3", "Q5J5C9", "Q30KP9", "Q8N687", "Q4QY38", "Q7Z7B8", "P59861", "Q8N690", "Q8NES8", "Q9H1M4", "Q8N688", "Q7Z7B7", "Q9BYW3", "Q8WTQ1", "P0DP74", "Q96PH6", "Q30KQ4", "Q30KQ5", "Q8IZN7", "Q30KQ9", "Q30KQ1", "Q30KQ3", "P0DP73", "Q8N104", "Q30KQ7", "Q8NG35", "Q30KQ6", "Q30KQ8", "Q8NET1", "O15263", "P81534", "P60022", "A8MXU0", "Q30KR1", "P51684", "P41597", "Q14643", "Q14573", "Q14571", "O43865", "Q96BZ4", "Q8IV08", "O14939", "Q8NEB5", "Q5VZY2", "P19174", "P20963", "P09693", "P08637", "P12314", "P43405", "P27986", "O00459", "P42336", "P42338", "Q02156", "O60733", "P63261", "P60709", "Q92747", "O15144", "P61158", "O15143", "P59998", "O15145", "O00401", "P42768", "P60953", "P53667", "P16333", "Q9NZQ3", "P62993", "O43516", "Q8TF74", "A6NGB9", "Q9Y2A7", "Q9NYB9", "Q8IZP0", "Q92558", "Q9UPY6", "Q9Y6W5", "Q8WUW1", "Q96F07", "Q9UQB8", "P35579", "Q9Y4I1", "Q13459", "O00159", "Q9UKX2", "P52735", "Q9UKW4", "P15498", "P00519", "Q13153", "P35240", "P23528", "Q9HD67", "Q92556", "Q96JJ3", "P46108", "Q14185", "Q05397", "P06241", "P12931", "P07947", "P07948", "P08631", "P14314", "P52907", "P47755", "P60900", "P25789", "P25788", "P60896", "O75832", "P25786", "P62333", "P62195", "P43686", "P62191", "P28065", "P28062", "A5LHX3", "Q14997", "P28072", "Q8TAA3", "Q92530", "P61289", "P28074", "Q9UL46", "P28070", "Q06323", "O00233", "P49720", "P48556", "P49721", "P40306", "Q16401", "P55036", "O14818", "Q9BXN2", "O95999", "Q9UDY8", "P01584", "P49427", "O15530", "Q9BXL7", "P0DP23", "P16298", "Q08209", "P63098", "Q13469", "Q12968", "O95644", "Q01201", "Q99558", "P61081", "Q8TBC4", "P01116", "P01116", "P01112", "Q9NNX6", "P04049", "P13598", "Q13177", "O75914", "P32942", "P22694", "P17612", "P22612", "Q9UMR7", "Q99IB8", "P04582", "P05877", "P05879",

"P05881", "P35961", "P19550", "P03377", "P04624", "P03375", "P12487", "Q70626", "P04581", "P05882", "P12490", "P12489", "P03378", "P20888", "P05880", "P19549", "P04580", "P12491", "P31872", "P05878", "P20871", "P19551", "P04583", "P31819", "P12488", "P18799", "P04579", "P98088", "Q6W4X9", "Q9H3R2", "Q5SSG8", "Q9H195", "Q7Z5P9", "Q02817", "Q8WXI7", "Q99102", "Q8TAX7", "Q8N307", "Q8N387", "P15941", "Q02505", "Q9HC84", "Q96DR8", "Q685J3", "Q9UKN1", "Q8IUN9", "Q9ULY5", "Q6EIG7", "Q96MN2", "Q9Y2E6", "Q9NSU2", "Q9H171", "Q13049", "Q9BRZ2", "P19474", "Q9UJV9", "P35222", "Q32MZ4", "Q8N884", "P42226", "Q7RTR2", "Q16666", "P78527", "P49959", "Q08211", "Q9H2U1", "P53803", "P61218", "P05423", "Q9NVU0", "P19388", "O14802", "P62875", "Q9NW08", "P0DPB5", "Q9BT43", "O15318", "Q9BUI4", "Q9H1D9", "Q9Y2Y1", "O15160", "O75575", "Q9Y535", "P52434", "O95944", "A8K4G0", "P43631", "Q496F6", "P13747", "Q13241", "P26717", "Q9NZC2", "P26718", "P06239", "Q07889", "P29353", "O43561", "O75791", "Q13094", "P43632", "Q9NP99", "Q14954", "Q14943", "A6NMB1", "Q6ZMC9", "Q14953", "Q9GZY6", "Q9UQC2", "P01854", "Q01362", "P12319", "Q04759", "Q7LDG7", "Q8TDF6", "O95267", "P42681", "P42680", "Q08881"]

UNIPROT (keyword:KW-0202) AND (reviewed:true) AND (taxonomy_id:9606)

Cytokines = ["O00175", "O00292", "O00585", "O00626", "O14625", "O14788", "O14793", "O15444", "O15467", "O43508", "O43557", "O43927", "O60383", "O60565", "O75610", "O75888", "O95150", "O95390", "O95393", "O95715", "O95760", "O95813", "O95972", "P01374", "P01375", "P01562", "P01563", "P01566", "P01567", "P01568", "P01569", "P01570", "P01571", "P01574", "P01579", "P01583", "P01584", "P02775", "P02776", "P02778", "P04141", "P05000", "P05013", "P05014", "P05112", "P05113", "P05231", "P06744", "P08700", "P09341", "P09603", "P09919", "P10145", "P10147", "P10451", "P10720", "P12643", "P12644", "P12645", "P13232", "P13236", "P13497", "P13500", "P13501", "P13725", "P14174", "P15018", "P15248", "P15514", "P16619", "P18075", "P19875", "P19876", "P20809", "P22003", "P22004", "P22301", "P22362", "P23510", "P24001", "P27539", "P28799", "P29459", "P29460", "P29965", "P32881", "P32970", "P32971", "P35225", "P40225", "P40933", "P41273", "P42830", "P43026", "P43490", "P47992", "P48023", "P48061", "P49771", "P50591", "P51671", "P55000", "P55107", "P55773", "P55774", "P58499", "P60568", "P78423", "P78556", "P80075", "P80098", "P80162", "Q06643", "Q07325", "Q12904", "Q13007", "Q14005", "Q14116", "Q14213", "Q16552", "Q16619", "Q16627", "Q16663", "Q6KF10", "Q6UWK7", "Q6UX27", "Q6UX46", "Q6UXT8", "Q6ZMJ4", "Q7Z4P5", "Q7Z5A7", "Q7Z5Y6", "Q86YJ6", "Q8IU54", "Q8IZ96", "Q8IZI9", "Q8NEV9", "Q8NHW4", "Q8TAD2", "Q8TAZ6", "Q8WWZ1", "Q92583", "Q969D9", "Q96DZ9", "Q96FZ5", "Q96MX0", "Q96PD4", "Q96S42", "Q99616", "Q99731", "Q99988", "Q9BXJ3", "Q9GZX6", "Q9H293", "Q9H2A7", "Q9H772", "Q9HBE4", "Q9NPF7", "Q9NPH9", "Q9NR23", "Q9NRJ3", "Q9NYY1", "Q9NZH6", "Q9NZH7", "Q9NZH8", "Q9P0W0", "Q9UBD3", "Q9UBD9", "Q9UBH0", "Q9UBR5", "Q9UHA7", "Q9UHD0", "Q9UK05", "Q9UNG2", "Q9Y258", "Q9Y275", "Q9Y4X3", "P05015", "P34820", "Q1L6U9", "Q6UX52", "Q86WN2", "Q8IZJ0", "Q8IZV2", "Q96QR1", "Q9P0M4", "Q9UHF5", "Q6EBC2"]

Id: Lipids R-HSA-556833.9 + O95445 (ApoM) + O43866 (CD5L - lipid synthesis)

Lipids = ["O95445", "O43866"]+["Q96PD7", "Q6ZNB7", "Q96PD6", "Q3SYC2", "Q86VF5", "Q9BQK8", "Q92539", "Q14693", "O75907", "Q14410", "Q14409", "P32189", "Q6NUI2", "Q9HCL2", "Q05469", "P41247", "O60664", "P15090", "Q99685", "O60240", "Q8WTS1", "P43304", "P36873", "P62136", "P62140", "Q7Z6Z6", "P17612", "P22694", "P22612", "Q03135", "Q0Z7S8", "O15540", "P12104", "P07148", "P51161", "P05413", "A6NFH5", "Q01469", "P02774", "O60494", "P11473", "Q07973", "Q99538", "O15528", "Q6VVX0", "Q8N2W9", "P63279", "P61956", "Q5SW96", "P98164", "P08185", "P80365", "P28845", "P26439", "P14060", "P05093", "P08686", "P19099", "P15538", "P01189", "P31213", "Q9H8P0", "Q53GQ0", "P37058", "P01215", "P01229", "P18405", "C9JRZ8", "P11511", "P14061", "Q9BPX1", "Q8NBQ5", "P37059", "P30536", "O95153", "P49675", "P10109", "Q6P4F2", "P05108", "P22570", "Q96DR4", "P59095", "Q14849", "O95772", "P15121", "P08842", "Q9Y6L6", "Q9Y2P5", "Q14032", "Q12908", "P19793", "Q15788", "Q96RI1", "Q15596", "O15438", "Q14973", "P46721", "O95342", "Q86UW2", "Q86UW1", "P02768", "Q9NPD5", "Q9NSY2", "P22680", "O75881", "Q9H1P3", "Q9BZF3", "Q9H4L5", "Q96SU4",

"Q9BZF2", "Q9BXW6", "P22059", "P22307-1", "P51659", "Q02318", "O14975", "Q9UHK6", "O14734", "P42330", "P52895", "Q04828", "P17516", "P51857", "Q9H2F3", "Q16647", "Q9UNU6", "Q99424", "Q9Y6A2", "Q9NYL5", "O95992", "Q15126", "P04035-2", "P04035-1", "Q15392", "P36956", "Q12772", "Q16850", "P53602", "Q9H2C2", "Q9UBM7", "O75845", "Q15125", "Q14739", "Q15800", "P37268", "O95749", "Q15738", "Q01581", "P14324", "Q03426", "Q9BXS1", "Q13907", "Q9BWD1", "P56937", "O76062", "P48449", "Q14534", "Q8IY26", "P36956-3", "P36956-1", "Q14974", "O43462", "Q12770", "Q9Y6B6", "P53992", "O94855", "O95486", "O95487", "Q15436", "Q9Y5U4", "O15503", "Q14703", "P62826", "P23511", "P25208", "Q13952", "P08047", "Q13085", "P49327", "Q9BZK7", "Q9BYK8", "Q3L8U1", "Q96RS0", "Q92793", "Q14686", "Q6STE5", "Q86X55", "Q15648", "Q07869", "O60907", "O00767", "O00763", "Q14872", "Q9H5J4", "Q96AQ7", "O60543", "A5D6W6", "Q8N6M3", "Q9Y5L2", "Q7Z5P4", "P55809", "Q9BYC2", "Q02338", "P24752", "Q8TB92", "P35914", "Q86V21", "Q9H6R3", "Q9BUT1", "P54868", "O75182-1", "O75376", "Q9Y618", "O15379", "Q96ST3", "P13196", "P11474", "Q16656", "Q86YN6", "P11310", "Q14192", "Q15067-1", "Q9NZI5", "Q6PCB7", "P50416", "P35398", "Q09472", "O75192", "Q16881", "Q02928", "P02647", "O60427", "P48163", "O15516", "Q99743", "O00327", "P27469", "O60656", "Q6Q788", "P21439", "O95477", "Q13133", "P28702", "P55055", "Q13227", "P04798", "P35869", "P27540", "Q9HBZ2", "A9YTQ3", "Q96RU7", "Q8IVF5", "O75509", "P20393", "Q99541", "P23786", "P48060", "Q06520", "Q9BY76", "P37231", "Q9UBK2", "Q9P086", "P24863", "O75586", "Q9Y2X0", "Q9NWA0", "O43513", "Q9Y3C7", "Q9H944", "Q6P2C8", "Q9BUE0", "Q96RN5", "O60244", "P49336", "A0JLT2", "Q9NX70", "Q9ULK4", "Q15528", "Q9UHV7", "Q9H204", "Q9BWU1", "O95402", "O75448", "Q13503", "Q96HR3", "Q9NVC6", "Q9NPJ6", "Q93074", "Q71F56", "Q96G25", "Q9BTT4", "Q71SY5", "Q9Y2W1", "Q9Y6Q9", "Q96EK7", "P01019", "Q9NZL6", "P02652", "P16671", "Q15327", "P33121", "Q9NUN7", "Q8TDN7", "P51648-1", "O14494", "P43353", "P48448", "Q5QJU3", "Q8IWX5", "Q9BX95", "O43688", "O14495", "O95470", "P33527", "Q9UNQ0", "P27544", "Q6ZMG9", "Q8IU89", "Q9HA82", "Q96G23", "Q8N5B7", "Q9NRA0", "Q06136", "Q8NHU3", "Q53FV1", "Q8N138", "Q9P0S3", "Q9NUV7", "Q8NFR3", "Q969W0", "O15269", "O15270", "O95292", "Q9P0L0", "Q5SGD2", "Q9Y5P4-2", "Q9NYA1", "Q7L5A8", "O43169", "P78368", "Q96LT4", "O15121", "Q15139", "O94806", "Q9BZL6", "Q6QHC5", "Q8IVW8", "A6NFX1", "Q86VZ5", "P07602", "Q6UWV6", "P17405", "P54803", "P06280", "Q8IW92", "Q6UWU2", "Q8NCI6", "P16278", "Q9NR71", "P15289", "P07686", "P06865", "P17900", "Q9NY59", "O60906", "P20645", "Q8NBK3", "Q8NBJ7", "Q13510", "Q9UQ49", "P04062", "Q9NXE4", "Q99519", "P10619", "Q8WWR8-2", "Q9Y3R4", "Q96EG1", "Q5FYB0", "P15848", "P51689", "P51690", "P54793", "Q5FYA8", "Q6UWY0", "Q5FYB1", "Q9H227", "Q9HCG7", "Q99999", "Q9NPC4", "O96024", "Q00973", "Q969X2", "Q9BVH7", "Q16842", "Q11203", "Q9BYG0", "P19526", "Q10981", "Q9UNP4", "Q16880", "O75752", "O15466", "Q9UBX8", "O43286", "Q8TCT0", "Q16739", "P50897", "Q9NV23", "Q4G176", "Q5K4L6", "Q5FVE4", "Q96GR2", "O95573", "O60488", "Q9HB03", "A1L3X0", "Q9BW60", "Q9GZR5", "Q5HYJ1", "Q9NZ01", "Q9ULC5", "Q9UKU0", "Q9NXB9", "Q9NYP7", "Q5VWC8", "Q9P035", "B0YJ81", "Q6Y1H2", "P86397", "P53007", "Q9UMR5", "P53396", "Q9Y6X9", "Q8N4T8", "Q92506", "Q86SK9", "Q15165", "Q15166", "P27169", "P20292", "P09917", "Q16873", "P18283", "P36969", "P07203", "P05177", "Q16678", "Q7Z449", "P78329", "P10632", "P11712", "P33261", "Q6GMR7", "Q5TCH4", "Q6NT55", "P13584", "Q9HBI6", "P98187", "Q08477", "P16050", "Q9H4B8", "Q9H4A9", "P16444", "P36269", "P19440", "P09960", "P49137", "Q14914", "P18054", "P47712", "Q8N8N7", "P35354", "P41222", "P24557", "Q8TBF2", "P15428", "O14684", "P23219", "P16152", "Q15185", "Q9H7Z7", "O60760", "P51589", "P34913", "O15296", "O00519", "Q9BYJ1", "O75342", "Q58HT5", "Q8NC06", "Q5T8D3", "Q15067-2", "O75521-2", "Q08426", "Q9NUI1", "O95822-2", "P33897", "Q8N9L9", "Q3I5F7", "P09110", "Q9NYQ3", "O43808", "O14832", "Q9BY49", "P51648-2", "Q9UJ83", "Q9NUZ1", "O15254", "P43155", "Q9UKG9", "P0C024", "A8MXV4", "P22307", "O95864", "Q8N6N7", "P07108", "Q96CM8", "Q709F0", "Q8IVS2", "O14561", "Q9Y305", "P49753", "Q8N1Q8", "Q5T1C6", "Q9BV79", "P30084", "P40939", "P55084", "Q16836", "P28330", "P16219", "P49748", "Q6P461", "Q53FZ2", "P42126", "Q16698", "P22033", "Q8IVH4", "P05165", "P05166", "Q96PE7", "Q6JQN1", "Q6ZUV0", "O00154", "Q8WXI4", "Q8WYK0", "Q9NPJ3", "Q86TX2", "Q9UKL6", "Q9BR61", "P42765", "O76082", "Q9UGJ0", "P54646", "O43741", "Q92523", "Q03181", "Q9NPA3", "Q92748",

"O43772", "Q6ZWT7", "Q6P1A2", "Q643R3", "Q6ZNC8", "P14555", "Q9NP80", "Q8TB40", "Q3MJ16", "P53816", "Q6P4A8", "Q68DD2", "P0C869", "Q86XP0", "O60733", "Q9UNK4", "O15496", "Q9NZ20", "P04054", "Q9BZM2", "Q9BZM1", "Q9NZK7", "P39877", "Q13018", "Q9UP65", "Q96KN8", "Q9HDD0", "Q9UL19", "Q9NWW9", "Q9NQZ5", "Q8TCT1", "P06276", "P22303", "Q8NA29", "Q8WU67", "Q8NF37", "Q9Y365", "Q9Y6K0", "P35790", "Q9Y259", "P28329", "Q8WUD6", "P68400", "P19784", "P67870", "Q9UBM1", "Q8IWA5", "Q53GD3", "Q8N4M1", "Q8NCS7", "Q8WWI5", "Q9Y5K3", "P49585", "Q9NPB8", "Q9H0X9", "Q9BZF1", "Q9BXB5", "Q53H76", "Q9NUQ2", "P09923", "O15228", "Q9NRZ5", "Q86UL3", "Q6UWP7", "Q99943", "O15120", "Q53EU6", "Q9NRZ7", "Q8N335", "P21695", "Q8N2A8", "Q8NAN2", "Q7L4E1", "O14939", "Q13393", "Q9NPH0", "Q6XZB0", "Q8WWY8", "Q8NEL9", "O94830", "P48739", "P48651", "Q9BVG9", "O14735", "Q92903", "Q9BZ71", "O00562", "Q9BZ72", "Q7L5N7", "Q8N661", "Q6P1J6", "Q9UJA2", "Q9NST1", "Q96AD5", "Q6ZPD8", "Q6E213", "Q16635", "Q8IV08", "Q96BZ4", "O95674", "Q8WUK0", "Q32NB8", "Q9NVF9", "Q9HBU6", "Q99447", "Q9C0D9", "Q8TBG4", "Q9UG56", "Q92604", "Q96N66", "Q53H12", "Q8NCC3", "Q99829", "O75131", "O95741", "Q9UBL6", "Q9NXD2", "Q13614", "Q9C0I1", "Q9NYA4", "Q13496", "Q9Y2I7", "Q92562", "Q08AM6", "Q9Y2H2", "O00443", "Q8NEB9", "Q99570", "Q96PE3", "O15327", "Q9BTU6", "Q8TCG2", "Q96QG7", "Q9Y216", "P42356", "P61204", "P84077", "Q9UBF8", "O75747", "P56180", "Q6XPS3", "Q9NRR6", "Q01968", "Q9NTJ5", "P78356", "Q8TBX8", "P48426", "Q86T03", "Q86WG5", "O14986", "Q99755", "Q12923", "Q9HB21", "Q9HB19", "Q9Y217", "Q9BT40", "Q15735", "Q92835", "O15357", "Q8NCE2", "O43426", "Q13615", "O15056", "Q13613", "P60484", "O60331", "Q9HB20", "Q96JA3", "Q9HAU0", "Q9Y2H5", "Q9H4M7", "Q96T51", "P42336", "O00459", "Q92569", "P42338", "Q8WYR1", "P48736", "O00329", "Q5UE93", "O00750", "P27986", "Q96EF0", "P51813", "P20338", "P61106", "P20339", "Q8WTR4", "Q8N9F7", "Q8IY17", "Q6UWR7", "Q9NZC3", "Q7L5L3", "Q6ZV29", "O95248", "Q8WVP5", "O95379", "Q6P589", "Q5GJ75", "O00116", "Q6IAN0", "Q96K12", "Q8WVX9", "P36969-2", "Q03013", "P05181", "P10635", "P08684"]

Semi-code for functional classification

Hemostasis = list(set (Hemostasis))
Cytokines = list(set (Cytokines)- set(Coagulation))
Complement Cascade = list(set (Complement Cascade)- set(Cytokines + Coagulation))
Immune Adaptive = list (set (Immune Adaptive) - set(ComplementCascade + Cytokines + Coagulation))
Other Immune-related = list(set (Immune Innate) - set (Immune Adaptive + ComplementCascade + Cytokines + Coagulation))
Orphans = is not one of the above
